# Resveratrol treatment reduces expression of MCP‐1, IL‐6, IL‐8 and RANTES in endometriotic stromal cells

**DOI:** 10.1111/jcmm.16178

**Published:** 2020-12-15

**Authors:** Roya Kolahdouz‐Mohammadi, Farzad Shidfar, Sepideh Khodaverdi, Tahereh Arablou, Sahel Heidari, Nesa Rashidi, Ali‐Akbar Delbandi

**Affiliations:** ^1^ Department of Nutrition School of Public Health Iran University of Medical Sciences Tehran Iran; ^2^ Endometriosis Research Center Iran University of Medical Science Tehran Iran; ^3^ Department of Immunology School of Medicine Iran University of Medical Sciences Tehran Iran; ^4^ Immunology Research Center Institute of Immunology and Infectious Diseases Iran University of Medical Sciences Tehran Iran

**Keywords:** ectopic, endometriosis, MCP‐1, IL‐6, IL‐8, RANTES, resveratrol, stromal cells

## Abstract

Endometriosis is an inflammatory disease affecting reproductive‐aged women. Immunologic disturbance, as well as inflammation, have crucial roles in the pathogenesis of endometriosis. In this study, we evaluated the effects of resveratrol treatment on expression of monocyte chemotactic protein‐1 (MCP‐1), interleukin‐6 (IL‐6), IL‐8, and regulated upon activation, normal T cell expressed and secreted (RANTES) in endometrial stromal cells from patients with endometriosis compared with non‐endometriotic controls. Thirteen eutopic (EuESCs) and nine ectopic (EESCs) endometrial stromal cells from endometriotic patients as well as eleven endometrial stromal cells from non‐endometriotic controls (CESCs) were treated with resveratrol (100 μmol/L) or ethanol, and gene and/or protein expression of MCP‐1, IL‐6, IL‐8 and RANTES was examined at 6, 24 and 48 hours following treatment in the cells from all origins. Resveratrol treatment significantly reduced gene and protein expression of MCP‐1, IL‐6, and IL‐8 in EuESCs and EESCs compared with CESCs (*P < *.05‐.001, *P < *.05‐.001 and *P < *.05‐<.01, respectively), and this reduction was more noticeable in EESCs than EuESCs (*P* < .05‐<.001). Besides, resveratrol treatment significantly reduced RANTES protein expression in EESCs in all time intervals (*P* < .05). Resveratrol treatment significantly reduced the expression of MCP‐1, IL‐6, IL‐8 and RANTES in EESCs.

## 1 INTRODUCTION

Endometriosis is an enigmatic gynaecological disease characterized by ectopic implantation of endometrial like tissues in extra‐uterine sites.^1^ It afflicts approximately 10% of reproductive‐aged women, 2%‐11% of asymptomatic women, and almost half of the women experiencing chronic pelvic pain and associated with dysmenorrhoea, dyspareunia, pelvic pain and infertility.^1^, ^2^ The pathogenesis of endometriosis is not precisely understood.^3^ Among numerous theories proposed to elucidate the pathophysiology of endometriosis, peritoneal implantation of viable endometrial cells during retrograde menstruation is the generally accepted one.^4^ However, retrograde menstruation occurs in almost all reproductive‐aged women, but only 10%‐20% of them develop endometriosis, so other factors must be involved in implantation and survival of the displaced endometrial cells.^5^ Based on recent findings, immune dysregulation in the peritoneal microenvironment and chronic inflammation have crucial roles in endometriosis development^6^ as increased levels of pro‐inflammatory cytokines and chemokines such as interleukin‐6 (IL‐6), IL‐8, monocyte chemotactic protein‐1 (MCP‐1), and regulated upon activation, normal T cell expressed and secreted (RANTES) have been detected in peritoneal fluid (PF) of endometriotic patients compared to non‐endometriotic participants, suggesting that these secretory products may be involved in endometriosis initiation and progression through the promotion of growth, adhesion, invasion and proliferation of endometrial cells.^7^, ^8^, ^9^

Current treatment modalities for endometriosis are surgical and hormonal treatments, but the high incidence of disease recurrence and side effects of these therapies limit their usage in a long period.^10^ Thus, in recent years, there has been an increasing emphasis on finding naturally occurring compounds for the management of endometriosis.

Resveratrol (trans‐3,5,4′‐trihydroxystilbene), a nutraceutical found in significant amounts in red grapes, berries, peanuts and red wine, is one of these substances.^11^ Protective effects of resveratrol on various diseases have been widely investigated in preclinical and clinical studies and attributed to anti‐oxidative, anti‐inflammatory, anti‐tumorigenic, anti‐atherogenic and anti‐ageing properties of resveratrol.^12^

The first animal study of the effect of resveratrol on endometriosis was reported by Bruner‐Tran et al in 2011.^13^ In that study, resveratrol treatment decreased the number and volume of endometriotic lesions in a nude mouse model of endometriosis. In subsequent studies, resveratrol treatment in animal models of endometriosis decreased the number and size of endometriotic implants and showed anti‐inflammatory, anti‐angiogenic, anti‐proliferative, anti‐oxidative, and pro‐apoptotic activities^12^ and in just one in vitro study resveratrol treatment showed anti‐inflammatory effect through suppression of IL‐8 expression in endometriotic stromal cells.^14^

So in this study, for the first time, we sought to investigate and compare the effect of resveratrol treatment on MCP‐1, IL‐6, and IL‐8 gene expression and protein production and RANTES protein expression in ectopic and eutopic endometrial stromal cells of endometriotic women (EESCs and EuESCs, respectively) and non‐endometriotic control endometrial stromal cells (CESCs).

## 2 MATERIALS AND METHODS

### 2.1 Patient recruitment and tissue collection

This study included fifty‐five patients admitted to gynaecology ward of Rassoul Akram hospital, who were allocated to two groups based on laparoscopy or hysterectomy findings: Group I (endometriosis group) consisted of forty women with stage III‐IV peritoneal endometriosis, and group II (control group) consisted of fifteen women with benign gynaecological diseases and no evidence of endometriosis.

All women enrolled were at reproductive age (19‐45 years old), with regular menstrual cycles, and were at the proliferative phase of the menstrual cycle. Those who had received hormonal treatment or antioxidant supplements within the last three months before sampling, or had the pelvic inflammatory disease, adenomyosis, malignancy, or were pregnant and breastfeeding were excluded. The diagnosis of endometriosis was initially evaluated by an expert clinician during laparoscopy and then confirmed by histopathological examination, and the severity of endometriosis was identified according to the revised American Society for Reproductive Medicine (rASRM).^15^ Before enrolling in the study, informed consent was obtained from each woman using protocols approved by the Human Ethics Committees of the Iran University of Medical Sciences (Code: IR.IUMS.REC.1395.28108).

Ectopic and eutopic endometrial samples were obtained through laparoscopic sampling and biopsy curette, respectively. Endometrial tissues were collected in sterile Falcon tubes containing Dulbecco's modified Eagle's medium (DMEM)‐F12 (Gibco, Grand Island, NY, USA) and 1% penicillin‐streptomycin antibiotics (Gibco, Grand Island, NY, USA) and immediately transported to the laboratory on ice, and a portion of tissue was sent to pathology for confirmation of endometriosis.

### 2.2 Isolation, culture and purification of endometrial stromal cells (ESCs)

Isolation, culture and purification of ESCs described previously.^16^ Briefly, fresh endometriotic tissue was minced to pieces of about 1 mm^3^ and digested in DMEM‐F12 containing 100 U/mL penicillin and 100 µg/mL streptomycin (Gibco, Grand Island, NY, USA), 2 mg/mL of type I collagenase (Sigma‐Aldrich, St Louis, MO, USA) and 300 µg/mL of deoxynuclease I (Takara, Tokyo, Japan) for 120 minutes at 37°C, with intermittent vortexing every 15 minutes. After this procedure, the cell suspension was passed through 100‐mm mesh (BD Biosciences, San Jose, CA, USA) to separate the cells from any remaining undigested tissues. The obtained single cells were transferred to T25 flasks and cultured under standard culturing conditions in DMEM‐F12 supplemented with 10% fetal bovine serum (FBS) (Gibco, Grand Island, NY, USA) and 1% penicillin‐streptomycin antibiotics (Gibco, Grand Island, NY, USA) in an incubator at 37°C in a humidified atmosphere with 5% CO2 for 24 hours. After this time, non‐adherent cells were removed by gentle washing, leaving purified adherent ESCs to reach 80% confluence. Some samples were excluded from the study due to inconsistent pathology reports, culture contamination, or absence of enough cell growth, especially in the case of EESCs. Finally, thirteen eutopic and nine ectopic endometrial tissues from endometriotic patients and eleven eutopic endometrial tissues from non‐endometriotic patients were used in this study. To confirm the purification of the ESCs, immunofluorescent staining and flow cytometry analysis were used for the following antibodies: vimentin, nestin, cytokeratin, CD10, CD44, CD73, CD105, CD34 and CD45. Based on our findings, the ESCs of all three origins were pure.^16^

### 2.3 Determining the appropriate concentration for treatment of ESCs with resveratrol

In a pilot study, various concentrations of resveratrol (Sigma‐Aldrich) (12.5, 25, 50, 100, 200 and 400 µmol/L) were tested to find safety dose of resveratrol by MTT assay,^17^ based on MTT assay findings,^16^ four samples of EuESCs were treated with 25, 50 and 100 µmol/L resveratrol and ethanol (vehicle) for 48 hours to determine appropriate treatment dose of resveratrol. For this purpose, the EuESCs were diluted with DMEM‐F12 containing 5% FBS to a seeding density of 16 × 10^4^ cells/well and seeded into 24‐well tissue culture plates (SPL Life Sciences, Korea) in a final volume of 1000 μL/well. After the cells were incubated for 3 hours, the EuESCs were treated with varying concentrations (25, 50 and 100 μmol/L) of resveratrol or ethanol. After 1 hour, lipopolysaccharide (LPS) (100 ng/mL) (Sigma‐Aldrich, St Louis, MO, USA)^18^ was added, and cells were cultured for 48 hours. The supernatant was then collected and stored at −40°C until assayed. The concentrations of IL‐6 and IL‐8 in the culture supernatants were determined in triplicate using commercially available IL‐6 and IL‐8 ELISA kits (BD Bioscience, San Diego, CA, USA). Based on ELISA test results, concentrations of IL‐6 and IL‐8 were more decreased in the presence of 100 µmol/L resveratrol (data not shown). Besides, some previous studies showed anticarcinogenic effects of resveratrol at doses 5 µmol/L and often close to 100 µmol/L.^19^ So this concentration of resveratrol was used in subsequent treatments.

### 2.4 Treatment of the ESCs with resveratrol

ESCs of three origins were seeded in 12‐well plates (SPL Life Sciences, Korea) at a density of 32 × 10^4^ cells/well and incubated for 3 hours. After 3 hours, ESCs were treated with 100 µmol/L resveratrol or ethanol, and 1 hour later, LPS (100 ng⁄mL) (Sigma‐Aldrich, St Louis, MO, USA)^18^, ^20^ was added, and cells were cultured for 6, 24 and 48 hours.

### 2.5 RNA isolation, complementary DNA (cDNA) synthesis and quantitative real‐time PCR (qRT‐PCR)

Total RNA was isolated from ESCs using Trizol reagent (Qiagen, Germany) in accordance with the manufacturer's instructions. Concentration and purity of the extract were measured at an absorbance wavelength of 260/280 using a NanoDrop 2000 spectrophotometer (Thermo Fisher Scientific, Waltham, MA, USA), and the RNA integrity and quality were assessed by electrophoresis on 1% agarose gel. The concentrations of RNA samples ranged from 319.00 to 1244.7 ng/µL, and A260/280 ratio was greater than 1.8. After this step, equal amounts of RNA (1 µg) extracted from each sample was reverse transcribed into cDNA by Revert Aid First Strand cDNA Synthesis Kit (Thermo Fisher Scientific, Waltham, MA, USA) according to the manufacturer's protocols. The quantitative reverse‐transcription polymerase chain reaction was performed in duplicate using Rotor‐Gene 3000 (Corbett Research, Sydney, Australia) with the syber premix Extaq (Biofact, Daejeon, Korea). The sequences of the primers, the size of amplicons and annealing temperature of each primer are shown in Table 1. A total reaction system of 20 μL contained syber premix Extaq (Biofact, Daejeon, Korea), 10 μL; primer pairs, 1 μL; cDNA template, 1 μL; and DNase‐free water, 8 μL. Glyceraldehyde‐3‐phosphate dehydrogenase (GADPH) was used as a housekeeping gene to normalize the amount of total RNA present in each reaction. Thermocycler conditions included an initial holding at 95°C for 15 minutes, followed by 40 cycles of 95°C for 20 seconds, annealing and elongation for 40 seconds and the melting step was from 60° to 99°C. Melting curve analyses were performed after amplification cycles to ensure the purity and specificity of the products. The efficiency of qRT‐PCR reaction was determined by the standard curve, which was derived from serial dilutions of cDNA and qRT‐PCR product in triplicate. The PCR amplification efficiency of these candidate genes ranged from 95% to 97%, and the regression coefficient (*R* ^2^ value) of the standard curve ranged from 0.968 to 0.998, well within the acceptable range of qRT‐PCR.^21^

**TABLE 1:** The quantitative real‐time PCR primers used in this study

Target gene	Accession No.	Primer sequence (5' to 3')	Amplicon size (bp)	Annealing temperature (°C)
MCP‐1	NM_002982.4	F: GAAAGTCTCTGCCGCCCTT	84	60
		R: TTGATTGCATCTGGCTGAGCG		
IL‐6	NM_001371096	F: CTATGAACTCCTTCTCCACAAGCGCCTT	127	62
	NM_000600.5	R: GGGGCGGCTACATCTTTGGAATCTT		
IL‐8	NM_000584.4	F: CTGCGCCAACACAGAAATTATTGTA	170	62
	NM_001354840.2	R: TTCACTGGCATCTTCACTGATTCTT		
GAPDH	NM_002046.7	F: GCACCGTCAAGGCTGAGAAC	138	58
	NM_001256799.3	R: TGGTGAAGACGCCAGTGGA		

Abbreviations: bp, base pair; F, forward; GAPDH, glyceraldehyde‐3‐phosphate dehydrogenase; IL‐6, interleukin‐6; IL‐8, interleukin‐8; MCP‐1, monocyte chemotactic protein‐1; R, reverse.

### 2.6 Measurement of MCP‐1, IL‐6, IL‐8 and RANTES proteins

The concentration of the cytokines MCP‐1, IL‐6, IL‐8 and RANTES in the supernatant of cultured ESCs was performed by ELISA, using commercially available kits (Duoset; R&D Systems, Minneapolis, MN, USA for MCP‐1 and RANTES and BD Bioscience, San Diego, CA, USA for IL‐6 and IL‐8) in parallel and duplicate in accordance with the manufacturer's instructions. The minimum detection levels for MCP‐1, IL‐6, IL‐8 and RANTES were 15.6, 4.7, 3.1 and 15.6 pg/mL, respectively.

### 2.7 Statistical analysis

The statistical analysis of data was carried out using the GraphPad Prism software (version 6). The Kolmogorov‐Smirnov test was used for the assessment of data normality. Based on the non‐parametric distribution of data, Wilcoxon and Mann‐Whitney U tests were used for comparison of variables within and between groups, respectively. For the comparison of multiple groups, Kruskal‐Wallis test with Dunn post hoc analysis was used. The analysis of gene expression was performed comparing the fold change and relative expression by calculating the 2^−ΔΔct^ and 2^−Δct^, respectively. Statistical significance was considered as *P* < .05.

## 3 RESULTS

### 3.1 The basal gene/protein expression levels of MCP‐1, IL‐6, IL‐8 and RANTES in ESCs

**FIGURE 1:**
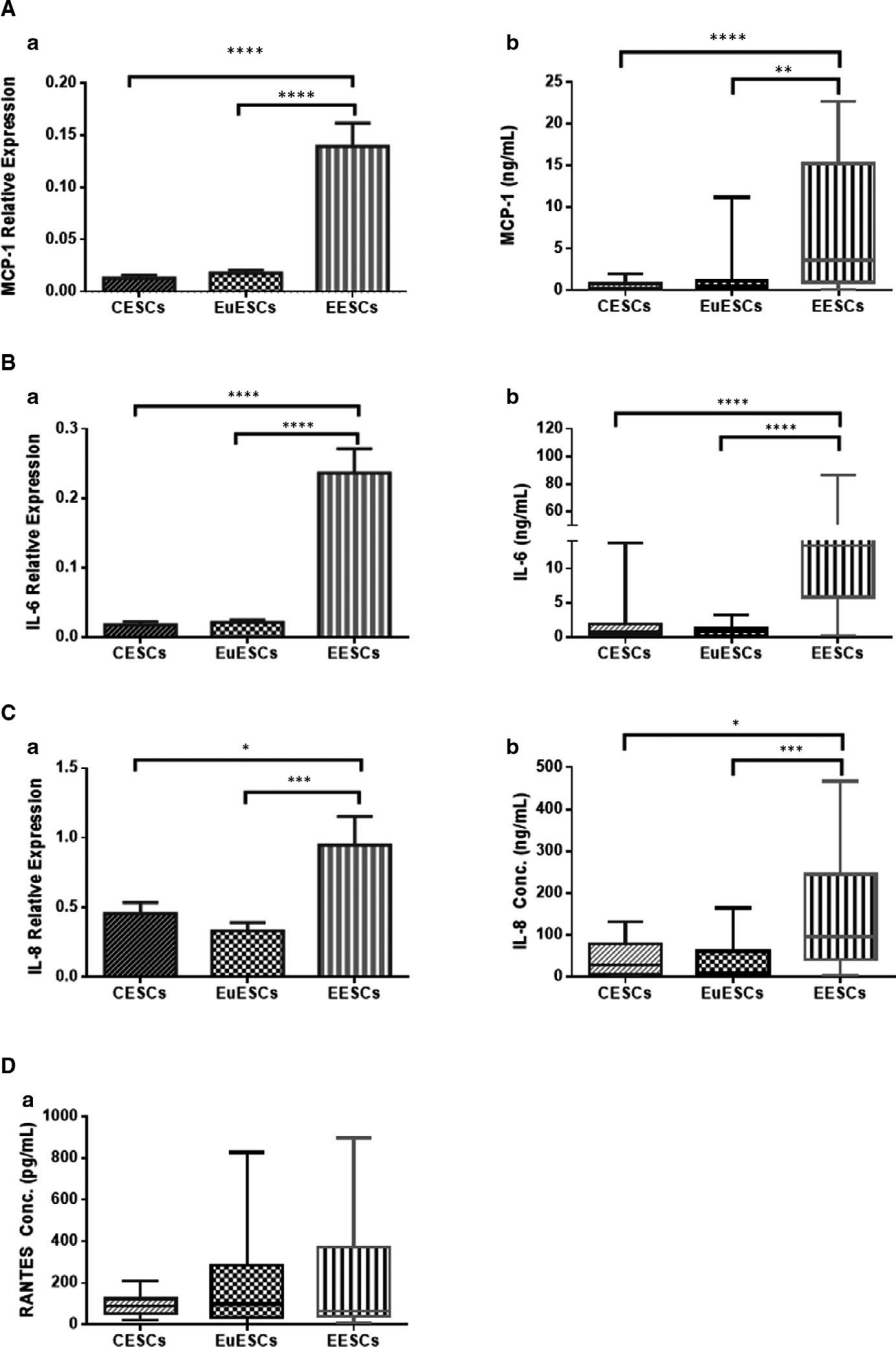
The basal expression levels of MCP‐1 (A), IL‐6 (B), IL‐8 (C), and RANTES (D) genes and/or proteins in ESCs. Expression levels of MCP‐1 mRNA (Aa), MCP‐1 protein (Ab), IL‐6 mRNA (Ba), IL‐6 protein (Bb), IL‐8 mRNA (Ca), IL‐8 protein (Cb) and RANTES protein (Da) were measured in CESCs from non‐endometriotic controls (n = 11), EuESCs (n = 13) and EESCs (n = 9) from endometriotic patients. Data were represented as mean ± SEM. Data from the basal protein expression were presented as box and whiskers graphs. *P < .05, **P < .01, ***P < .001, ****P < .0001. CESCs, control endometrial stromal cells; EESCs, ectopic endometrial stromal cells; ESCs, endometrial stromal cells; EuESCs, eutopic endometrial stromal cells; MCP‐1, monocyte chemotactic protein‐1; RANTES, regulated upon activation, normal T cell expressed and secreted

Based on our findings, the basal gene and protein expression of MCP‐1, IL‐6 and IL‐8 were significantly higher in EESCs compared with EuESCs and CESCs (*P* < .01‐<.0001; *P* < .0001; and *P* < .05‐<.001, respectively). RANTES protein expression was also higher in EESCs compared with CESCs, but the difference was not significant (Figure 1).

### 3.2 The effect of resveratrol treatment on MCP‐1 gene and protein expression in ESCs

**FIGURE 2:**
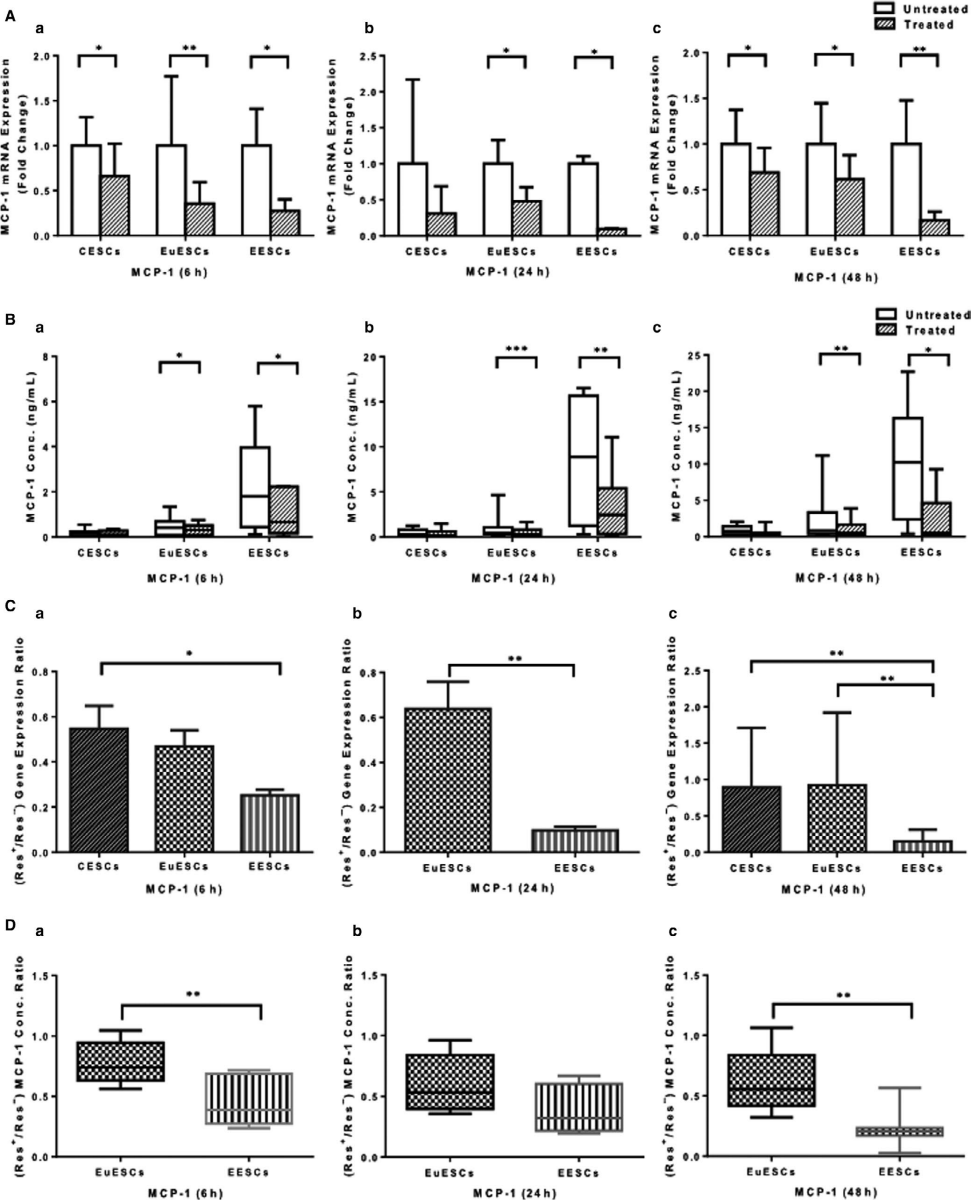
The effect of resveratrol treatment on MCP‐1 gene and protein expression in ESCs. CESCs from non‐endometriotic controls (n = 11), EuESCs (n = 13) and EESCs (n = 9) from endometriotic patients were cultured in the presence or absence of resveratrol for 6, 24 and 48 hours. MCP‐1 gene (A) and protein (B) expression at 6 (a), 24 (b) and 48 (c) hours after treatment were measured by quantitative real‐time PCR and enzyme‐linked immunoassay, respectively. The differential effect of resveratrol treatment on ESCs from different groups was calculated as the ratio of the gene (C) and protein (D) expression of MCP‐1 in the presence and absence of resveratrol at 6 (a), 24 (b) and 48 (c) hours. Data were represented as mean ± SEM. Data from the protein expression were presented as box and whiskers graphs. *P < .05, **P < .01 and ***P ≤ .001. CESCs, control endometrial stromal cells; EESCs, ectopic endometrial stromal cells; ESCs, endometrial stromal cells; EuESCs, eutopic endometrial stromal cells; MCP‐1, monocyte chemotactic protein‐1

As shown in Figure 2A, resveratrol treatment reduced MCP‐1 gene expression in EESCs and EuESCs at 6, 24 and 48 hours (*P* < .05‐<.01) and in CESCs at 6 and 48 hours (*P* < .05). Regarding MCP‐1 protein expression, resveratrol treatment reduced MCP‐1 protein expression in EESCs and EuESCs at 6, 24 and 48 hours (*P* < .05‐.001) and had no effect on CESCs (Figure 2B). The reducing effect of resveratrol treatment on MCP‐1 gene expression was only significant in EESCs compared with EuESCs and CESCs in all time intervals (*P* < .05‐<.01) (Figure 2C), and as shown in Figure 2D, the reducing effect of resveratrol treatment on MCP‐1 protein expression was more remarkable in EESCs than EuESCs at 6 and 48 hours (*P* < .01).

### 3.3 The effect of resveratrol treatment on IL‐6 gene and protein expression in ESCs

**FIGURE 3:**
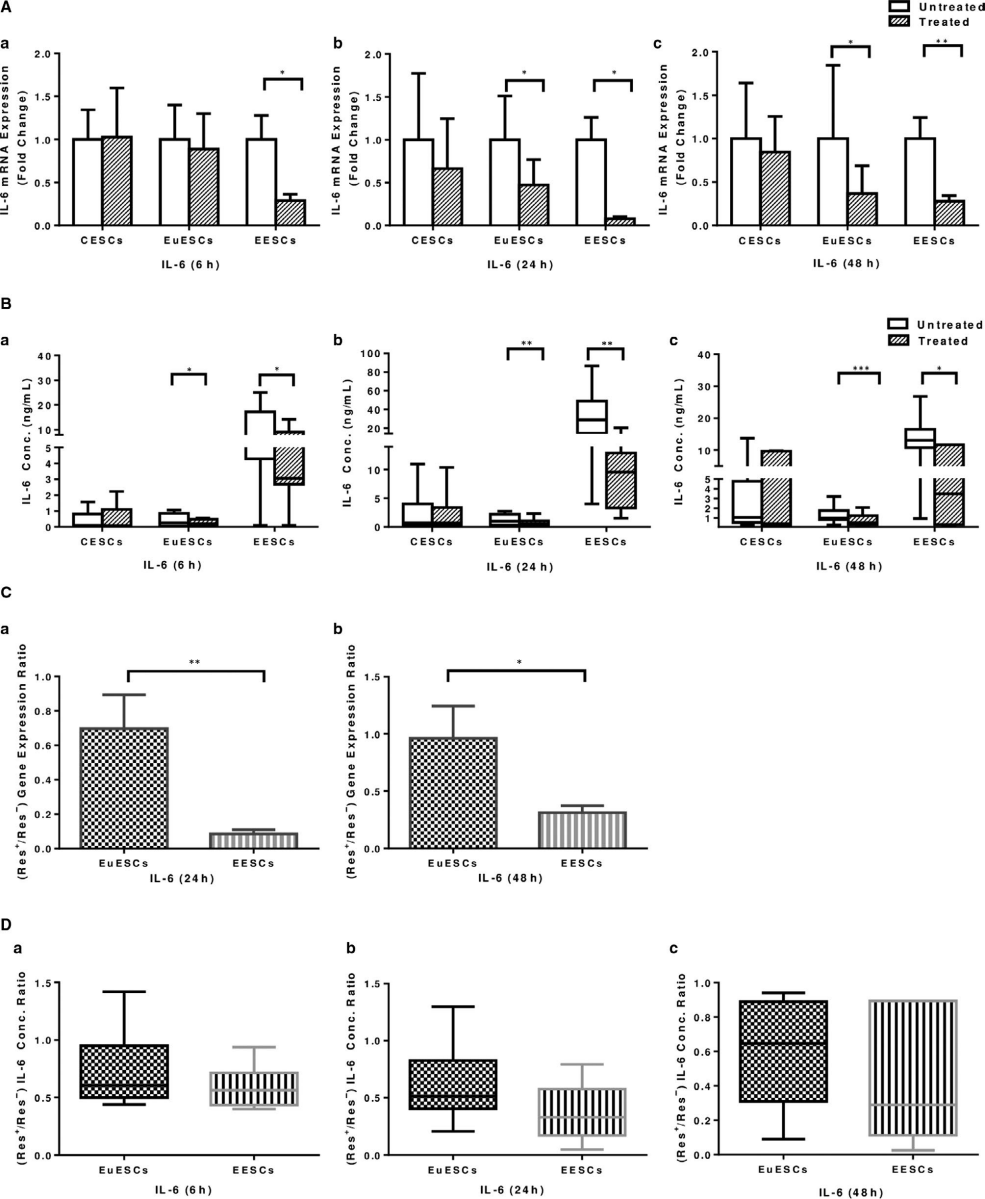
The effect of resveratrol treatment on IL‐6 gene and protein expression in ESCs. CESCs from non‐endometriotic controls (n = 11), EuESCs (n = 13) and EESCs (n = 9) from endometriotic patients were cultured in the presence or absence of resveratrol for 6, 24 and 48 hours. IL‐6 gene (A) and protein (B) expression at 6 (a), 24 (b) and 48 (c) hours after treatment were measured by quantitative real‐time PCR and enzyme‐linked immunoassay, respectively. The differential effect of resveratrol treatment on EuESCs and EESCs was calculated as the ratio of the gene (C) and protein (D) expression of IL‐6 in the presence and absence of resveratrol at 6 (a), 24 (b) and 48 (c) hours. Data were represented as mean ± SEM. Data from the protein expression were presented as box and whiskers graphs. * *P* < .05, **P < .01 and *** *P* ≤ .001. CESCs, control endometrial stromal cells; EESCs, ectopic endometrial stromal cells; ESCs, endometrial stromal cells; EuESCs, eutopic endometrial stromal cells

As shown in Figure 3A, resveratrol treatment reduced IL‐6 gene expression in EESCs at 6, 24 and 48 hours (*P* < .05‐<.01) and in EuESCs at 24 and 48 hours (*P* < .05) and had no effect on CESCs. Regarding IL‐6 protein expression, resveratrol treatment reduced IL‐6 protein expression in EESCs and EuESCs at 6, 24 and 48 hours (*P* < .05‐.001) and had no significant effect on CESCs (Figure 3B). The effect of resveratrol treatment on IL‐6 gene expression reduction was more significant in EESCs compared with EuESCs in all time intervals (*P* < .05‐<.01; Figure 3C). However, the differential effect of resveratrol treatment on IL‐6 protein expression reduction was not statistically significant between EuESCs and EESCs at 6, 24 and 48 hours (Figure 3D).

### 3.4 The effect of resveratrol treatment on IL‐8 gene and protein expression in ESCs

**FIGURE 4:**
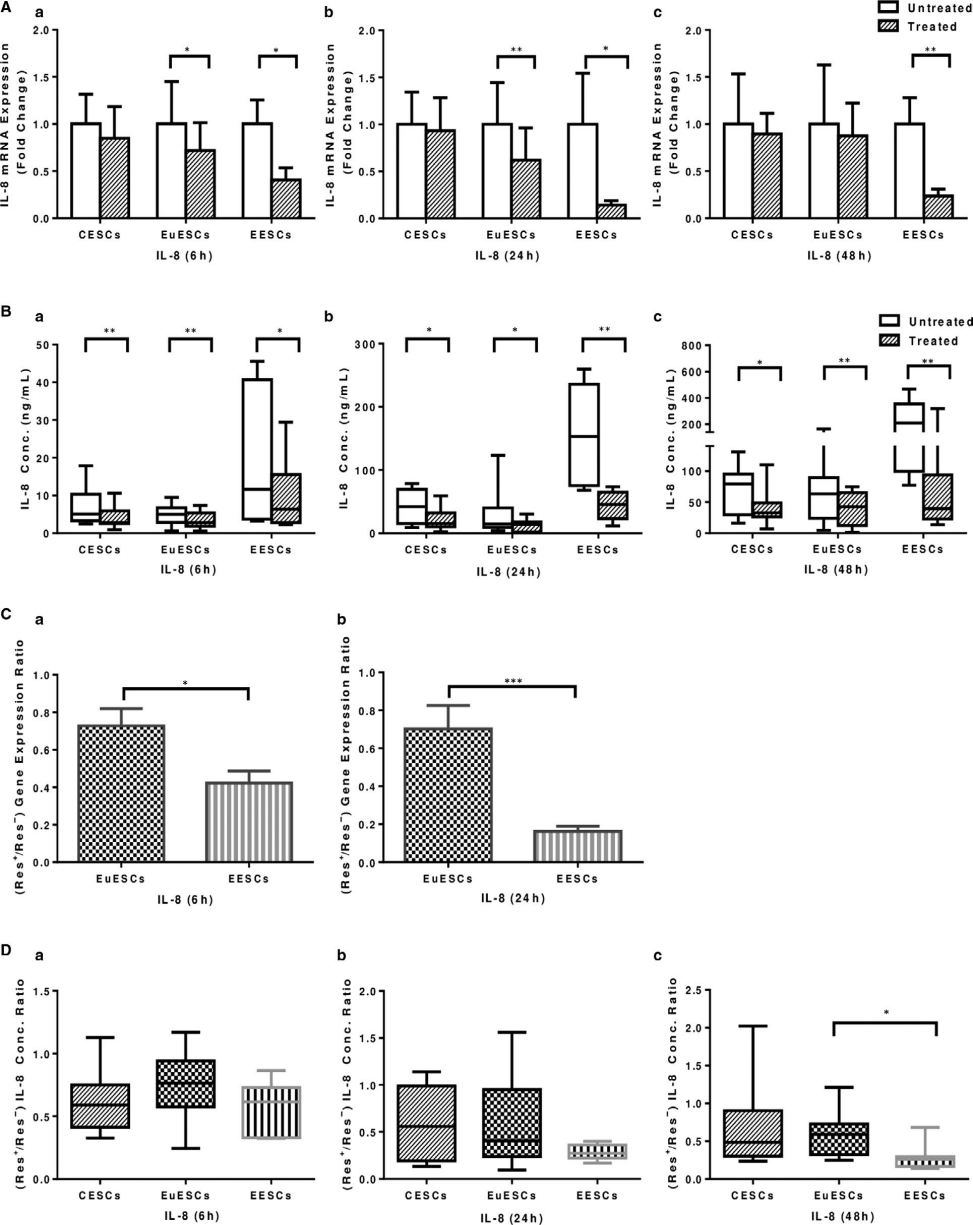
The effect of resveratrol treatment on IL‐8 gene and protein expression in ESCs. CESCs from non‐endometriotic controls (n = 11), EuESCs (n = 13) and EESCs (n = 9) from endometriotic patients were cultured in the presence or absence of resveratrol for 6, 24 and 48 hours. IL‐8 gene (A) and protein (B) expression at 6 (a), 24 (b) and 48 (c) hours after treatment were measured by quantitative real‐time PCR and enzyme‐linked immunoassay, respectively. The differential effect of resveratrol treatment on ESCs from different groups was calculated as the ratio of the gene (C) and protein (D) expression of IL‐8 in the presence and absence of resveratrol at 6 (a), 24 (b) and 48 (c) hours. Data were represented as mean ± SEM. Data from the protein expression were presented as box and whiskers graphs. *P < .05, **P < .01 and ***P < .001. CESCs, control endometrial stromal cells; EESCs, ectopic endometrial stromal cells; ESCs, endometrial stromal cells; EuESCs, eutopic endometrial stromal cells

Resveratrol treatment reduced IL‐8 gene expression in EESCs at 6, 24 and 48 hours (*P* < .05‐<.01) and in EuESCs at 6 and 24 hours (*P* < .05‐<.01) and had no effect on CESCs (Figure 4A). Besides, resveratrol treatment reduced protein expression of IL‐8 in EESCs, EuESCs and CESCs at 6, 24 and 48 hours (*P* < .05‐<.01, Figure 4B). The effect of resveratrol treatment on IL‐8 gene expression reduction was more significant in EESCs compared with EuESCs at all time intervals (*P* < .05‐<.001, Figure 4C), although the differential effect of resveratrol treatment on IL‐8 protein expression reduction was only significant at 48 hours in EESCs compared with EuESCs (*P* < .05, Figure 4D).

### 3.5 The effect of resveratrol treatment on RANTES protein expression in ESCs

**FIGURE 5:**
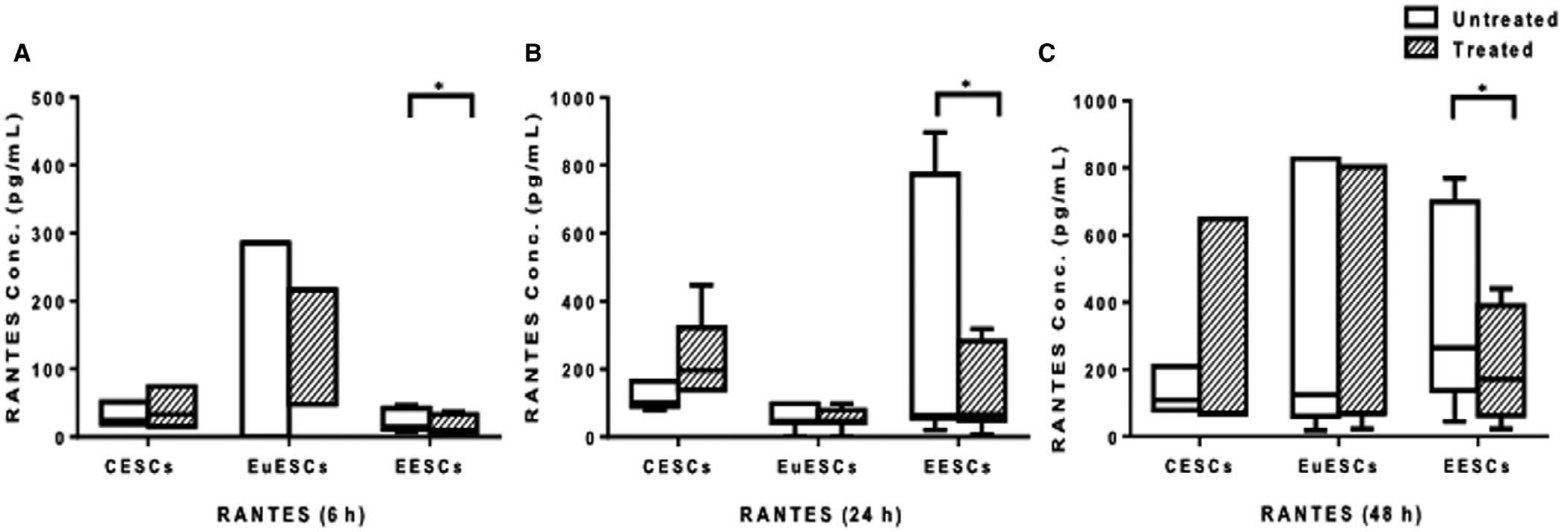
The effect of resveratrol treatment on RANTES protein expression in ESCs. CESCs from non‐endometriotic controls (n = 11), EuESCs (n = 13) and EESCs (n = 9) from endometriotic patients were cultured in the presence or absence of resveratrol for 6, 24 and 48 hours. RANTES protein expression at 6 (a), 24 (b) and 48 (c) hours after treatment was measured by enzyme‐linked immunoassay. Data were presented as box and whiskers graphs. *P < .05. CESCs, control endometrial stromal cells; EESCs, ectopic endometrial stromal cells; ESCs, endometrial stromal cells; EuESCs, eutopic endometrial stromal cells; RANTES, regulated upon activation, normal T cell expressed and secreted

Resveratrol treatment reduced RANTES protein expression in EESCs at 6, 24 and 48 hours (*P* < .05) and had no effect on EuESCs and CESCs (Figure 5).

## 4 DISCUSSION

We demonstrated in this study that EESCs express higher levels of MCP‐1, IL‐6 and IL‐8 under basal conditions than EuESCs and CESCs. RANTES protein expression was also higher in EESCs than EuESCs and CESCs, but the difference was not significant. To the best of our knowledge, only one study compared the in vitro production of MCP‐1 by ESCs in patients with and without endometriosis.^22^ Consistent with our findings, in that study, EuESCs secreted more MCP‐1 than CESCs. Regarding IL‐6 and IL‐8, our observations are well in accordance with previous studies that reported increased gene and/or protein expression of IL‐6^23^, ^24^, ^25^ and IL‐8^23^ in EESCs than EuESCs or CESCs. Regarding RANTES, previous studies also showed higher protein secretion by EESCs compared to CESCs.^26^, ^27^

Sampson's theory (retrograde menstruation) is one of the most accredited hypotheses in explaining the pathophysiology of endometriosis.^4^ As retrograde menstruation occurs in most cycling women, but only a minority develop endometriosis so additional factors like oxidative stress, inflammation and immunologic changes may contribute to the development of endometriosis.^6^ Increased levels of reactive oxygen species (ROS) and pro‐inflammatory cytokines in PF of endometriotic patients have been reported in a number of studies.^7^, ^28^ Excessive production of ROS as a result of iron overload as well as pro‐inflammatory cytokines and LPS have been shown to activate the nuclear factor kappa B (NF‐κB) pathway, and NF‐κB further increases transcription of multiple genes encoding pro‐inflammatory cytokines, chemokines, angiogenic, adhesion and growth factors that are known to be involved in development and progression of endometriosis.^29^ Besides, constitutive activation of NF‐κB has been demonstrated in endometriotic lesions and peritoneal macrophages of endometriotic patients compared to controls.^30^, ^31^ So activation of NF‐κB in endometriotic lesions may be an explanation for increased gene and protein expression of MCP‐1, IL‐6, IL‐8 and RANTES in EESCs compared to EuESCs and CESCs.

MCP‐1 and its receptor CCL2 have been shown to play a crucial role in the initiation and progression of endometriosis.^32^ MCP‐1 is produced by many cell types, including macrophages, fibroblasts, endothelial and endometriotic stromal cells.^33^, ^34^ and many studies have documented elevated levels of MCP‐1 in the PF of patients with endometriosis.^7^

MCP‐1 attracts monocytes and T lymphocytes.^35^, ^36^ Moreover, MCP‐1 has been demonstrated to increase apoptosis of T lymphocytes and not endometrial cells through an increase in Fas ligand (FasL) production in human ESCs.^37^ On the other hand, Li et al showed that expression of CCL2 in the EuESCs could enhance ESCs survival and invasion through activation of Akt and mitogen‐activated protein kinase/ extracellular signal‐regulated kinase 1/2 (MAPK/Erk1/2) signalling pathway as anti‐CCL2 neutralizing antibody or CCR2 antagonist can completely decrease these effects.^38^ Moreover, in our study, resveratrol treatment reduced gene and protein expression of MCP‐1 in EESCs and EuESCs. However, this reduction was more pronounced in EESCs compared to EuESCs and CESCs. To the best of our knowledge, no study was investigated the effect of resveratrol treatment on MCP‐1 gene and protein expression in ESCs but in only two studies resveratrol treatment in experimentally induced endometriosis rat model significantly reduced MCP‐1 PF levels in treated groups compared to controls.^39^, ^40^

IL‐6 is one of the most prominent pro‐inflammatory cytokines and angiogenic factors in endometriosis.^41^ It is strongly expressed by peritoneal macrophages^42^ and endometriotic lesions,^43^ and significantly increased IL‐6 levels have been reported in PF and endometriotic lesions of patients with endometriosis compared to non‐endometriotic controls.^7^, ^43^ Besides, in line with our findings, other studies have indicated increased IL‐6 production by EESCs than EuESCs or CESCs.^23^, ^24^, ^25^ IL‐6 is a crucial mediator of cytokine cascade in endometriosis.^44^ Besides, endometriotic stromal cell‐derived IL‐6 stimulates peritoneal macrophages towards M2‐polarization. This type of macrophages plays an important role in inflammation and angiogenesis and causes ectopic endometrium to escape from apoptosis.^45^ So IL‐6 might be one of the cytokines involved in the pathogenesis of endometriosis. So far, only one study investigated the effect of resveratrol treatment on plasma and PF levels of IL‐6 on an experimental rat model of endometriosis.^46^ Resveratrol treatment in that study significantly reduced plasma and PF levels of IL‐6 compared to control.^46^ In our study for the first time, resveratrol treatment reduced gene and protein expression of IL‐6 in EuESCs and EESCs and had no effect on CESCs.

IL‐8 is a chemokine with potent neutrophil and T cell chemotactic activities.^47^ Monocytes, macrophages,^48^ eutopic and ectopic endometrial stromal cells are principal sources of IL‐8.^23^ Inflammatory cytokines like IL‐1, tumour necrosis factor‐α (TNF‐α) and LPS can also affect the release of this chemokine.^49^, ^50^ Many studies have pointed to increased PF levels of IL‐8 and its correlation with disease stage.^51^ Besides consistent with our findings, other studies have shown increased IL‐8 production by EESCs than EuESCs or CESCs.^23^ IL‐8 may be involved in all processes related to the pathogenesis of endometriosis like adhesion, invasion, implantation and proliferation of ectopic endometrial cells. Besides, it may protect ectopic endometrial cells from apoptosis through stimulation of FasL apoptosis in activated T cells and inhibition of neutrophil apoptosis.^52^ Up to now, only one study investigated the effect of resveratrol treatment on plasma and PF levels of IL‐8 on an endometriosis rat model. Resveratrol treatment in that study significantly reduced plasma and PF levels of IL‐8 compared to control.^46^ Besides, in a study by Taguchi et al, resveratrol treatment significantly suppressed TNF‐α ‐induced IL‐8 release from the endometriotic stromal cells.^14^ In line with this finding, resveratrol treatment in our study significantly reduced gene and protein expression of IL‐8 in EESCs.

RANTES is a potent chemotactic factor for monocytes and activated T cells.^53^ RANTES is produced by endometriotic stromal cells and peritoneal macrophages,^54^ and its concentrations are elevated in the peritoneal cavity of endometriotic patients and correlate with the disease severity.^55^ In our study, RANTES secreted more by EESCs and EuESCs compared to CESCs; however, the difference in secretion was not significant. In line with our findings, previous studies showed higher RANTES protein expression by EESCs and EuESCs compared to CESCs.^26^, ^27^ RANTES can recruit inflammatory cells into the peritoneal cavity, and these cells, in turn, can release a variety of pro‐inflammatory cytokines and angiogenic factors.^56^ Besides, high levels of RANTES in the ectopic milieu can induce macrophages tolerant, and this tolerant, in turn, not only inhibits apoptosis but also enhances the growth of ESCs.^27^ On the other hand, IL‐1ß, as a predominant activated macrophage product, increases RANTES expression via NF‐κB in endometriotic stromal cells.^57^

In our study, resveratrol treatment significantly reduced protein expression of RANTES in EESCs, and to the best of our knowledge, no study was investigated the effect of resveratrol treatment on RANTES protein expression in ESCs.

The mechanisms of resveratrol action related to reduced MCP‐1, IL‐6, IL‐8 and RANTES expression in EESCs are probably through regulation of pathways involved in oxidative stress, inflammation, cyclooxygenase (COX)‐2 and Sirtuin 1 (Sirt1). Iron overload has been shown in different compartments of the peritoneal cavity of endometriotic patients and leads to the generation of ROS, which, along with a decrease in antioxidant defence in endometriotic patients result in oxidative stress.^28^ Oxidative stress also impairs cellular functions through regulation of NF‐κB,^29^ which is involved in endometriosis development. NF‐κB activation stimulates the synthesis of pro‐inflammatory cytokines (IL‐6, IL‐8, MCP‐1 and RANTES), which are implicated in endometriotic cell proliferation, invasion and angiogenesis^58^ so inhibition of NF‐κB activation may decrease the expression of these pro‐inflammatory cytokines in endometriotic stromal cells. Resveratrol, as an antioxidant, has been shown to block NF‐κB activation induced by ROS, TNF‐α, pro‐inflammatory cytokines and LPS in several cell types.^59^, ^60^ On the other hand, elevated levels of TNF‐α, IL‐1ß, IL‐6 and IL‐8 secreted by peritoneal macrophages and ectopic endometriotic lesions induce COX‐2 expression and prostaglandins E2 (PGE2) production.^61^ Based on the literature, eutopic endometrium of endometriotic patients express more COX‐2 than disease‐free women, and COX‐2 protein is highly expressed in ectopic than eutopic endometrium in endometriotic women.^62^ COX‐2 inhibition in animal studies prevented the establishment and maintenance of endometriosis and decreased size and number of endometriotic tissues.^63^ Besides, studies have shown that increased COX‐2 and COX‐2 derived PGE2 production regulate survival, migration and invasion of endometriotic stromal cells in humans.^62^ Regarding resveratrol, Murias et al concluded that hydroxylated resveratrol analogues are selective COX‐2 inhibitors.^64^ Besides, Sirt1, a mammalian homolog of silent information regulator 2 (Sir2), may be involved in endometriosis pathogenesis.^65^ Overexpression of Sirt1 was reported to suppress cytokines production and reduce inflammation in different animal models.^66^ Sirt1 inhibits NF‐κB activity and thereby inflammatory cytokine production.^67^ In a study by Taguchi et al, Sirt1 activation by resveratrol significantly suppressed TNF‐α‐induced IL‐8 release by endometriotic stromal cells and suppression of Sirt1 by sirtinol (an inhibitor of Sirt1) enhanced IL‐8 secretion by endometriotic stromal cells.^14^

Therefore, in the light of present and previous findings, we can speculate that resveratrol as a natural and safe treatment may slow down the development of endometriosis and ameliorate its manifestations through its pleiotropic properties. However, as adhesion molecules, extracellular matrix metalloproteinase and other pro‐inflammatory cytokines activate/alter peritoneal microenvironment and epigenetic as well as genetic mechanisms have role in endometriosis pathogenesis, future studies should aim to clarify the effect of resveratrol on these factors.^68^, ^69^

## 5 CONCLUSION

Our results showed that EESCs differed significantly from EuESCs and CESCs concerning MCP‐1, IL‐6, IL‐8, and RANTES gene and/or protein expression. Besides, resveratrol treatment in this study significantly reduced the expression of MCP‐1, IL‐6, IL‐8 and RANTES in EESCs. Based on the results presented here and beneficial effects of resveratrol treatment in animal models of endometriosis, further in vivo and randomized controlled trials are recommended to achieve more conclusive results about the efficacy of resveratrol in endometriosis treatment.

## CONFLICT OF INTEREST

None declared.

## AUTHOR CONTRIBUTIONS

**Roya Kolahdouz‐Mohammadi:** Conceptualization (equal); Data curation (equal); Formal analysis (equal); Investigation (equal); Methodology (equal); Project administration (lead); Writing‐original draft (lead). **Farzad Shidfar:** Conceptualization (equal); Methodology (equal); Writing‐review & editing (equal). **Sepideh Khodaverdi:** Conceptualization (equal); Writing‐review & editing (equal). **Tahereh Arablou:** Data curation (equal); Writing‐review & editing (equal). **Sahel Heidari:** Data curation (equal); Writing‐review & editing (equal). **Nesa Rashidi:** Data curation (equal); Writing‐review & editing (equal). **Ali‐Akbar Delbandi:** Conceptualization (equal); Formal analysis (equal); Methodology (equal); Visualization (equal); Writing‐review & editing (equal); Supervision (lead).

## Data Availability

All data supporting the findings of this study are available from the corresponding author on request.
